# Phylogenomics of strongylocentrotid sea urchins

**DOI:** 10.1186/1471-2148-13-88

**Published:** 2013-04-23

**Authors:** Kord M Kober, Giacomo Bernardi

**Affiliations:** 1 Department of Ecology & Evolutionary Biology, University Of California Santa Cruz, Santa Cruz, CA, USA; 2 Department of Physiological Nursing, University Of California San Francisco, San Francisco, CA, USA

**Keywords:** Strongylocentrotus, Phylogenomics, Sea urchin, Holarctic expansion, Bering Strait, Vicariant divergence

## Abstract

**Background:**

Strongylocentrotid sea urchins have a long tradition as model organisms for studying many fundamental processes in biology including fertilization, embryology, development and genome regulation but the phylogenetic relationships of the group remain largely unresolved. Although the differing isolating mechanisms of vicariance and rapidly evolving gamete recognition proteins have been proposed, a stable and robust phylogeny is unavailable.

**Results:**

We used a phylogenomic approach with mitochondrial and nuclear genes taking advantage of the whole-genome sequencing of nine species in the group to establish a stable (i.e. concordance in tree topology among multiple lies of evidence) and robust (i.e. high nodal support) phylogenetic hypothesis for the family Strongylocentrotidae. We generated eight draft mitochondrial genome assemblies and obtained 13 complete mitochondrial genes for each species. Consistent with previous studies, mitochondrial sequences failed to provide a reliable phylogeny. In contrast, we obtained a very well-supported phylogeny from 2301 nuclear genes without evidence of positive Darwinian selection both from the majority of most-likely gene trees and the concatenated fourfold degenerate sites: ((*P. depressus, (M. nudus, M. franciscanus*), (*H. pulcherrimus,* (*S. purpuratus,* (*S. fragilis,* (*S. pallidus,* (*S. droebachiensis, S. intermedius*)). This phylogeny was consistent with a single invasion of deep-water environments followed by a holarctic expansion by *Strongylocentrotus*. Divergence times for each species estimated with reference to the divergence times between the two major clades of the group suggest a correspondence in the timing with the opening of the Bering Strait and the invasion of the holarctic regions.

**Conclusions:**

Nuclear genome data contains phylogenetic signal informative for understanding the evolutionary history of this group. However, mitochondrial genome data does not. Vicariance can explain major patterns observed in the phylogeny. Other isolating mechanisms are appropriate to explore in this system to help explain divergence patterns not well supported by vicariance, such as the effects of rapidly evolving gamete recognition proteins on isolating populations. Our findings of a stable and robust phylogeny, with the increase in mitochondrial and nuclear comparative genomic data, provide a system in which we can enhance our understanding of molecular evolution and adaptation in this group of sea urchins.

## Background

Sea urchins are benthic marine echinoderms distributed across all of the world’s oceans [1]. Despite their unusual appearance, they have been a component of human diets since at least the ancient Greeks [2] and are still experiencing a vigorous fisheries industry today [3,4]. The contribution of sea urchins to our understanding of many aspects of basic biology cannot be understated [5]. Sea urchins are a primary research model for embryology [5], fertilization [6], bilaterian development [7], genomic regulatory systems [8,9] and our basic understanding of fundamental properties of genomes [10,11]. They provide broadly useful natural systems in which we investigate central evolutionary questions of natural selection [12,13], reproductive isolation [14] and speciation [15-17] and ecological questions of population responses to disease [18] and global scale habitat distribution patterns [19]. Indeed, our first coherent view of cancer was provided by studying embryonic development in sea urchins [20] and origins of the phagocytic theory, a key process in the idea of an immune system, were based on observations of the movement and engulfing of foreign particles in echinoderm tissue [21].

The location of Echinodermata as an early branch in the deuterostome phylogeny serves as an important node with which to infer ancestral states of vertebrate biology [22,23]. This placement is useful for addressing broad reaching questions on the origins and evolution of animal immunity [24] and development [25]. Among sea urchins, the family Strongylocentrotidae is arguably the best studied group [26] and includes the well-annotated genome of the representative model species *Strongylocentrotus purpuratus*[27]. The Strongylocentrotidae are abundant marine echinoids with members living in the northern Pacific, northern Atlantic and the holarctic regions [26]. The group is comprised of four genera: *Strongylocentrotus*, *Hemicentrotus*, *Pseudocentrotus* and *Mesocentrotus*[28].

The phylogenetic position of strongylocentrotids relative to other sea urchins is well understood [29-31]. The genus *Strongylocentrotus* comprises five species: *S. purpuratus*, *S. pallidus*, *S. droebachiensis*, *S. intermedius*, *S. fragilis* and *S. polyacanthus*. *Strongylocentrotus djakonovi* has been assigned as a junior synonym for *S. droebachiensis*[32]*, S. pulchellus* a junior synonym for *S. intermedius*[32] and *A. fragilis* is a junior synonym for *S. fragilis*[28]. *Mesocentrotus*[33] comprises *M. franciscanus* (nee *S. franciscanus*) and *M. nudus* (nee *S. nudus*). *Hemicentrotus* and *Pseudocentrotus* are monotypic with *H. pulcherrimus* and *P. depressus*, respectively.

Recent mitochondrial molecular phylogenies have identified two clades, one consisting of members of *Strongylocentrotus* and *Hemicentrotus* and the other consisting of *Mesocentrotus* and *Pseudocentrotus*[34-36]. However, the branching orders within the *Strongylocentrotus* and *Hemicentrotus* clades are largely incongruent. Specifically, the relationships of *S. intermedius*, *S. droebachiensis* and *S. pallidus*, the relative placements of *S. purpuratus* and *S. fragilis*, and the positions of *S. intermedius* and *H. pulcherrimus* are unresolved.

This issue is outstanding because an accurate and robust phylogeny is essential for correctly interpreting the broad range of contemporary biological research being performed on this group. The unresolved phylogenetic relationships among strongylocentrotids underscores the problem of using few loci in a group with large effective population sizes and complex histories that may involve hybridization [37]. This is particularly relevant in this group since sea urchins are broadcast spawners, where fertilization occurs in the water column. Many strongylocentrotid species live in sympatry, display overlapping spawning seasons, and have unequal gametic compatibilities [38]. The fertilization efficiencies of eggs and sperm between species are often asymmetric and gamete recognition loci are thought to play an important role in post-mating pre-zygotic isolation [39]. Selection on components of gamete interactions are thought to be particularly important early on in the speciation process. In Strongylocentrotidae, however, gametes from distantly related sympatric *M. franciscanus* and *S. purpuratus* readily fertilize in the lab, but hybrids are seldom seen in nature and no introgression has been observed between these two species [37]. Therefore, the rapid evolution of gamete recognition proteins is of particular interest in this group and is under intense study [13,14,40]. An accurate phylogeny is integral to this work.

The combined action of incomplete lineage sorting and introgression of genes between species are known to greatly complicate the resolution of species trees [41-43] weakening single loci phylogenetic inferences [44]. Congruence among multiple genes and morphology has been suggested as a robust approach to reconstruct a reliable phylogeny [45]. Multi-locus analyses at genome-wide scales offer a remarkable opportunity for powerful improvements in molecular phylogenetic inference [46]. The advent of next-generation sequencing and genome assembly makes such analyses possible. A high quality, well-annotated, draft genome for *S. purpuratus* is available [27,47,48] and high coverage, whole-genome sequencing, has been completed for nine of the ten species comprising the family Stron gylocentrotidae (Kober and G. H. Pogson, unpublished data).

The objective of this study was to establish a strong phylogenetic hypothesis for the family Strongylocentrotidae based on alignments of nuclear and mitochondrial genes from 9 (out of the 10) species of the family. The development of a robust and stable phylogeny in this group will provide essential comparative tools to a vast group of scientists including those interested in ecology, evolution, developmental biology and physiology.

## Results

### Mitochondrial DNA genome assemblies

All together, we obtained representative sequence from 9 of the 10 species in the strongylocentrotid group. We generated *de novo* assemblies of the complete mtDNA genome from five species of *Strongylocentrotus* and three additional members of the family (Table 1). With the exception of *S. pallidus*, we observed no changes in gene order or sequence inversions. This is consistent with what has been seen in other echinoids [49]. However, in our assembly of *S. pallidus*, we observed an inversion in the region flanking *ND3* through the middle of *ND5*, spanning *ND4*. As such, the reverse complement of *ND3* and *ND4* were included in our analysis. The disruption of the *ND5* sequence precluded using it in our analyses and for consistency the entire gene was excluded to keep all alignments comparable.

**Table 1:** Genera and species of sea urchins used in this study

**Genus**	**Species**	**Geographic range**	**Depth range (m)**	**Mitochondrial genome size**
*Strongylocentrotus*				
	*S. purpuratus* (Stimpson)	EP	0-160	-
	*S. pallidus* (Sars)	HA	5-1600	15,552
	*S. droebachiensis* (O. F. Müller)	HA	0-1150	15,046
	*S. intermedius* (A. Agassiz)	WP	0-225	15,718
	*S. fragilis* (Jackson)	EP	0-1150	15,748
*Mesocentrotus*				
	*M. franciscanus* (A. Agassiz)	EP	0-125	15,364
	*M. nudus* (A. Agassiz)	WP	0-180	15,628
*Hemicentrotus*				
	*H. pulcherrimus* (A. Agassiz)	WP	0-45	15,721
*Pseudocentrotus*				
	*P. depressus* (A. Agassiz)	WP	0-5	15,736

Geographic ranges: West Pacific (WP), East Pacific (EP), Holarctic (HA).

### Alignments and model selection

Details of the alignments, conserved block totals identified by Gblocks and parsimony-informative character totals are summarized in Table 2. The best-fitting model of nucleotide substitution was observed to vary considerably among mitochondrial genes. We selected *Paracentrotus lividus* to root the mitochondrial trees because it is strongly supported as an appropriate outgroup for Strongylocentrotidae [30,31,34,35,49]. The nuclear trees were rooted at the midpoint between the two major clades of strongylocentrotid [34,35]. This rooting was consistent with the topology of our MA and M4 mtDNA tree rooted with *P. lividus*, though caution is recommended as resolution of the phylogeny of members within this group using mtDNA genes is unreliable (this study).

**Table 2:** The data partitions used for phylogenetic analysis

**Dataset**		**Num.**	**Alignment length**		**Nuc. Sub.**	**MP**	**MP**
		**Genes**	**Full**	**Gblocks**	**Model**	**Const.**	**Inform.**
Mitochondrial							
	*12S*	1	917	879	TN93	-	-
	*16S*	1	1,566	1,509	TVM	-	-
	*ATPase6*	1	693	683	GTRG	-	-
	*ATPase8*	1	180	164	HKY85I	-	-
	*CytB*	1	1,142	1,142	TIMG	-	-
	*COI*	1	1,554	1,554	GTRG	-	-
	*COII*	1	690	690	TRNG	-	-
	*ND1*	1	972	972	F81	-	-
	*ND2*	1	1,067	1,057	GTRIG	-	-
	*ND3*	1	351	309	GTR	-	-
	*ND4*	1	1,395	1,377	GTR	-	-
	*ND4L*	1	300	294	GTRIG	-	-
	*ND6*	1	508	471	TRNIG	-	-
	MA	13	n/a	10,656	*FR*	6,826	2,158
	M4	11	1,075	-	*FR*	131	644
Nuclear							
	N4N	2301	297,856	-	*FR*	229,627	42,176
	N4S	879	163,484	-	*FR*	126,801	22,984
	N4A	3180	461,340	-	*FR*	356,428	65,160

The alignment length prior to Gblocks (Full), the resulting alignment size of conserved sites (Gblocks), the maximum parsimony constant (MP Const.) and informative (MP Inform.) sites are listed. Datasets including multiple genes include all Mitochondrial genes (MA), fourfold degenerate sites (4ds) of mitochondrial genes (M4), 4ds site of nuclear genes with no evidence of positive selection (N4N), 4ds sites of nuclear genes having evidence of positive selection (N4S) and 4ds sites of all nuclear genes (N4A). The nucleotide substitution models (Nuc. Sub. Model) are listed including the free rates mixture model implemented in PhyML (*FR*).

### Tests for molecular adaptation of mitochondrial genes

We found no evidence for positive selection acting on any of the protein coding mitochondrial genes tested (*ATPase6, COI, COII, CytB, ND1, ND2, ND3, ND4, ND4L*). Internal stop codons were found for at least one site in the alignments for *ND4* and *ND6* and the approximate likelihood calculation in *ATPase8* was unreliable. For these reasons we did not test these three genes for evidence of positive selection.

### Phylogenic relationships inferred from neutral nuclear genes

**Figure 1:**
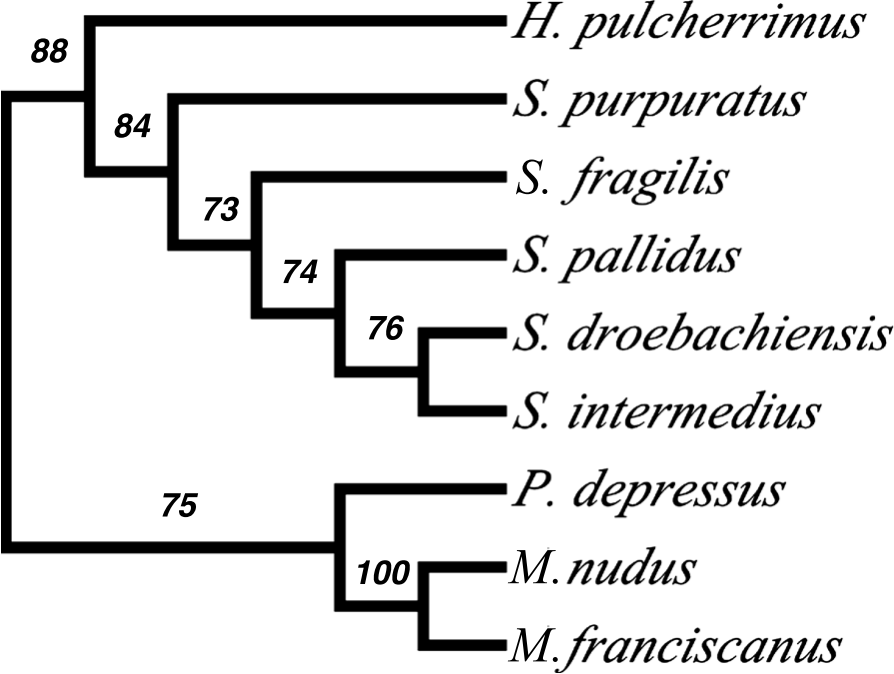
**The cladogram of the most frequent tree obtained from the Maximum Likelihood analysis of 2301 nuclear genes without evidence of positive selection.** Branch support is quantified as the frequency that the node is supported by a gene alignment where the most frequent tree was not rejected or the gene’s ML tree was significantly different from the most frequent tree (see text). The tree is rooted between the two major clades identified in this group.

Incomplete lineage sorting and introgression can cause difficulties in phylogenetic reconstruction [42,50]. This can be particularly troublesome in groups with short, rapid bursts of divergence. We collected the ML trees generated for alignments of 2301 nuclear neutral genes and identified the most frequent topology (Figure 1). We implemented the SH test to evaluate the support for this tree. For each gene, we tested whether its ML tree was significantly different than the most frequent ML tree. If the gene’s ML tree was not significantly different than the most frequent ML tree, then the latter was used as the representative tree for that gene for the frequency calculations of tree node support. We found the most frequent ML tree was supported and not significantly different from the gene’s ML tree for 69.23% (1593 of 2301) of nuclear genes having no evidence of positive selection (called here neutral genes) (Figure 1). The density tree of the most frequent tree and the ML gene trees that significantly differed from the most frequent tree are shown in Additional file 1: Figure S1.

**Figure 2:**
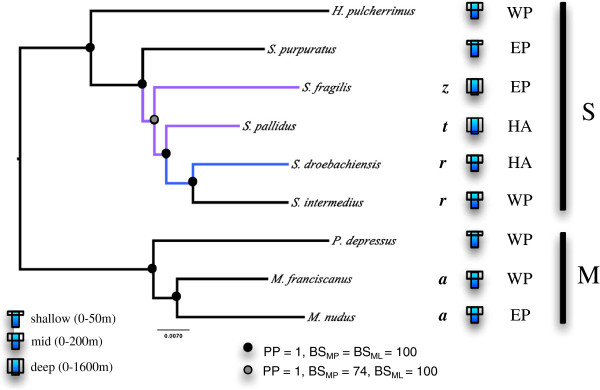
**The 50% majority rule consensus phylogram of the stationary trees obtained from the Bayesian inference analysis of concatenated neutral nuclear genes at four-fold degenerate sites mid-point rooted between the two major clades previously identified.** Branch support values are the BI posterior probabilities (PP), MP bootstrap (BS_MP_) and ML bootstrap (BS_ML_) for genes rejecting evidence of positive selection. Branches leading to deep water species are colored in purple. The branch leading to *S. droebachiensis* is colored blue, as this species occurs primarily in shallow water but can range to a depth of 300 m. Adult depth range: s, shallow (0-50 m); m, medium (0-200 m); d, deep (0-1600 m). Distributions: West Pacific (WP), East Pacific (EP), holarctic (HA). The cross-section of the ultrastructure of primary spines [59]: rectangular (*r*), trapezoid (*z*), triangular (*t*) or ansiform (*a*).

The inferred phylogenetic relationships of *Strongylocentrotus* are shown in Figure 2 by the phylogram generated from the concatenated fourfold degenerate sites of nuclear neutral genes. We define very strong support as having a BI posterior probability (PP) of ≥99, a ML bootstrap value (BS_ML_) of ≥97, and a MP bootstrap value (BS_MP_) of ≥97. The topology of the most frequent ML gene tree is identical to the MP 50% majority-rule consensus tree, the ML tree, and the BI 50% majority-rule consensus phylogram of the stationary tree inferred from fourfold degenerate sites of all nuclear genes regardless of whether the genes showed evidence of positive selection or not (N4A, N4S and N4S datasets) (Figure 2).

The BI 50% majority-rule consensus phylogram of the stationary tree inferred from fourfold degenerate sites of nuclear genes without selection (‘N4Ds tree’) had complete (BI PP = 1, BS_ML_ = BS_MP_ = 100) or very strong support from all three methods at all nodes with the N4N and N4A datasets except at the divergence of *S. fragilis* (BS_MP_ = 74 and BS_MP_ = 69, respectively). As such, we observed an effect on the phylogenetic inference when including genes found to be under positive selection. Indeed, the tree obtained from the N4S data produced a similar topography except *S. purpuratus* and *S. fragilis* branching locations were swapped, with *S. fragilis* the earlier branching of the two with low support for the node (not shown).

**Figure 3:**
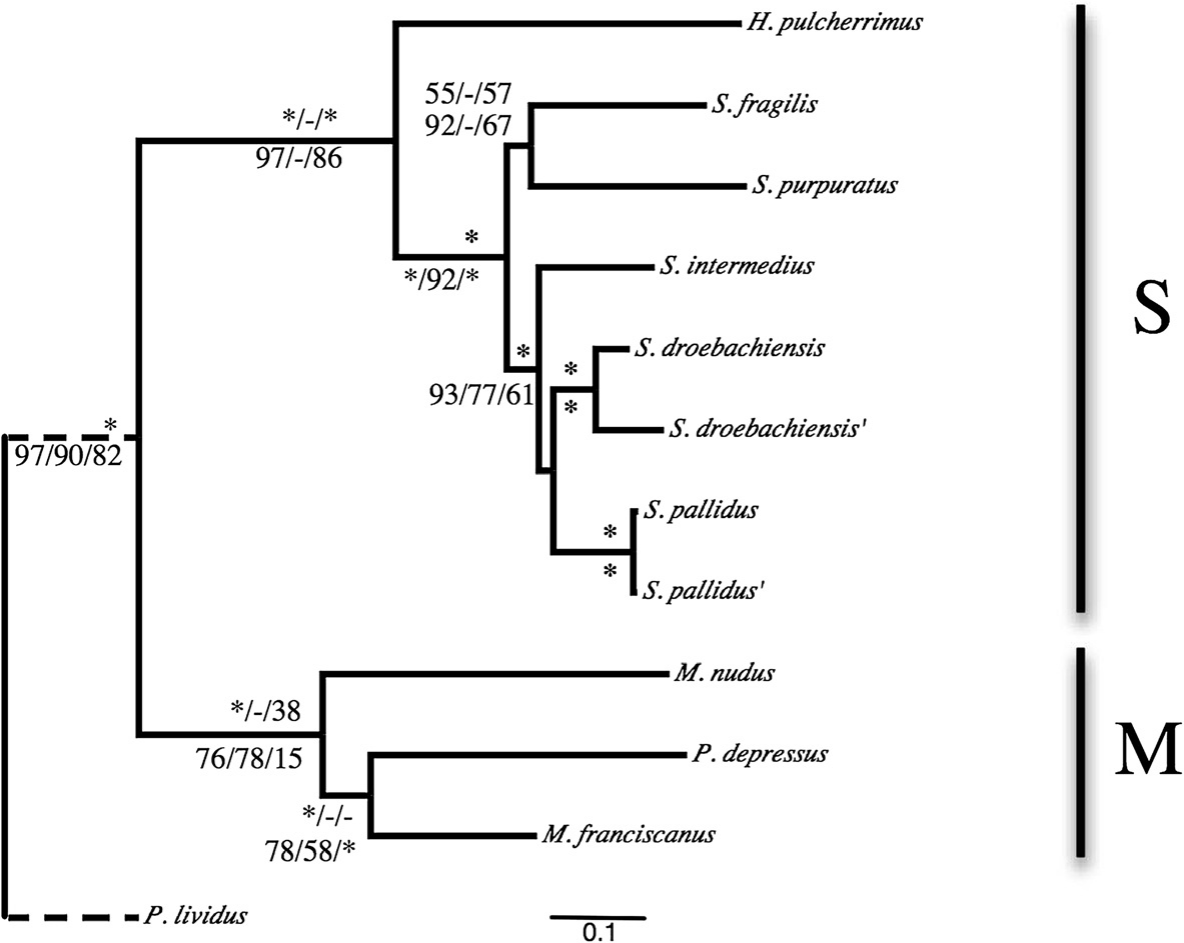
**The 50% majority rule consensus phylogram of the stationary trees obtained from the Bayesian inference analysis of concatenated mitochondrial genes at all sites.** Branch support are the Bayesian Inference posterior probabilities (BI PP), Maximum Parsimony bootstrap (MP BS) and Maximum Likelihood bootstrap (ML BS) for concatenated mitochondrial genes above and four-fold degenerate sites below the branch. Asterisks on the branch labels denote strong support for the method or all methods (BI PP > =99, MP BS > =95, ML BS > =95). Unsupported nodes are indicated with ‘-‘. Single quotation marks next to a taxon name denote the *de novo* assembled individual from this study of the species. Scale bar, substitutions per site.

We found strong support for the two major separate clades in Strongylocentrotidae in our analyses of concatenated nuclear (N4A, N4S and N4N) and mitochondrial data (MA, M4). Hereafter, we will refer to the major clade comprised of *M. nudus*, *M. franciscanus* and *P. depressus* as ‘clade M’. The remaining focal taxa (*Hemicentrotus* and *Strongylocentrotus*) form a monophyletic group we hereafter refer to as clade ‘S’ (Figure 2). Within clade M, the concatenated mitochondrial genes and nuclear genes exhibited different branching orders. The concatenated fourfold degenerate sites of nuclear genes, whether under positive selection or not, resolve *M. nudus* sister to *M. franciscanus* with very strong support. One the other hand, the BI and ML trees, but not the MP trees, of both MA and M4 datasets support *P. depressus* sister to *M. franciscanus* (Figure 3).

Comparing the mtDNA and nuclear results, we observed very strong support for *H. pulcherrimus* sister to the clade containing *S. fragilis*, *S. purpuratus*, *S. intermedius*, *S. pallidus* and *S. droebachiensis* across our analysis of concatenated datasets. We also found very strong support for a monophyletic clade of *S. intermedius*, *S. droebachiensis* and *S. pallidus* across our analysis of concatenated datasets. However, MA and M4 datasets produced *S. pallidus* sister to *S. droebachiensis*, but without strong support. In contrast, the N4A, N4S and N4N concatenated datasets found very strong support *S. intermedius* as sister to *S. pallidus*.

### Phylogenic relationships inferred from mitochondrial genes

In all mitochondrial ML gene trees except *ATPase8, 12S* and *ND6*, the *S. pallidus* and *S. droebachiensis* individuals from GenBank and our *de novo* assemblies resolved as sister taxa as expected. However, the putative outgroup, *P. lividus*, consistently produced a very long branch and that shifted to different locations among the ML gene trees. Ignoring a *P. lividus* root, the individual mitochondrial ML gene trees topologies were consistent in resolving Clade M and Clade S (Additional file 2: Figures S2, Additional file 3: Figure S3, Additional file 4: Figure S4). However, the branching order within these clades was inconsistent and in conflict with the nuclear data. We found contradictory topologies for the relative positions of *S. fragilis* and *S. purpuratus* among mitochondrial genes trees. *H. pulcherrimus* was placed sister to *Strongylocentrotus* species in all gene trees except *ATPase6*, *ND4L* and *ND6*. No single mitochondrial gene returned a topology corroborating with the N4Ds tree.

The locations of *S. purpuratus* and *S. fragilis* were discordant between the MP method and BI and ML methods in the MA and M4 datasets. BI and ML trees had these two species sister to *S. intermedius*, *S. pallidus* and *S. droebachiensis* (Figure 3), whereas the MP method has *S. purpuratus* branching earliest, then *S. fragilis* and then the *S. intermedius*, *S. pallidus* and *S. droebachiensis* observed with the nuclear concatenated datasets (not shown). The monophyly of a *S. intermedius*, *S. pallidus* and *S. droebachiensis* clade was recovered in both MA and M4, but we found conflicting support for a sister relationship between *S. pallidus* and *S. droebachiensis* versus *S. pallidus* and *S. intermedius* (Figure 3).

The *12S* sequences used by Lee (2003) were collected, aligned, and used to construct an ML tree as described above for rRNA mitochondrial genes. The proposed relationship shown in Figure 2 of Lee (2003) was found to be no better at explaining these data (*P* > 0.505) than our proposed species tree (Figure 1). The proposed tree of Lee (2003) differed significantly from our proposed species tree (Figure 1) in 762 of 2301 nuclear genes tested. The N4N tree was significantly better at explaining the data for 685 genes, while Lee (2003) Figure 2 was significantly better for 77. When we included the *12S* sequences of Lee (2003) with our *12S* data, our alignment and ML method produced a different tree (Additional file 5: Figure S5). Here, *H. pulcherrimus* and *S. nudus* individuals resolved as sister taxa, but *S. intermedius* falls in sister to the *S. pallidus* sequences rather than with the *S. intermedius* of Lee (2003). The *S. intermedius* sequence of Lee (2003) falls sister to *S. fragilis* in a clade with *S. purpuratus*.

The combined dataset (*COI*, *COII*, *tRNA-Lys*, *ATPase8* and *ATPase6*) of Biermann *et al.* (2003) was collected and concatenated after removing *S. polyacanthus*. An alignment was generated and ML trees reconstructed as described above for the protein-coding mitochondrial genes. The proposed relationship in Figure 2 of Biermann *et al.* (2003) was found to be significantly better at explaining these data (*P* < 0.001) than our proposed species tree (Figure 1). However, the proposed tree of Figure 2 of Biermann *et al.* (2003) differed significantly from our proposed species tree (Figure 1) in 1865 of 2301 nuclear genes tested. Our species tree was significantly better at explaining the data for 1855 genes, while Figure 2 of Biermann *et al.* (2003) was significantly better for 10 genes.

Using RNA secondary structure in phylogeny reconstruction has been shown to have significant utility in resolving relationships in metazoan taxa [51-53]. However, our results from *12S* and *16S* mixed model and un-partitioned analyses produced very similar trees (not shown).

### Rate of molecular evolution and divergence times

**Figure 4:**
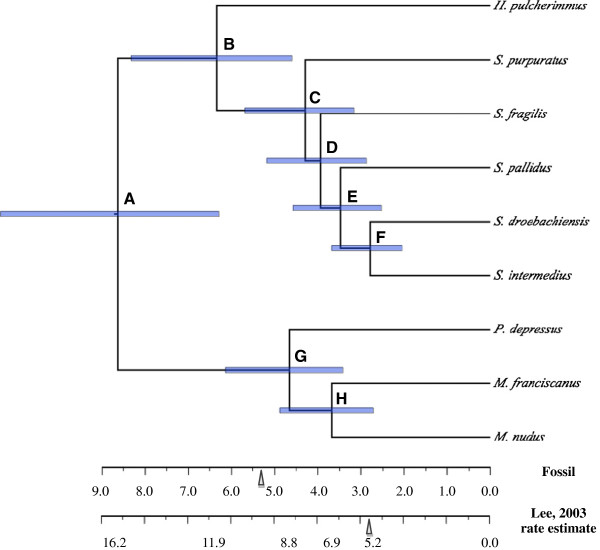
**The molecular clock enforced dated phylogram from Bayesian Inference (BI) among fourfold degenerate sites from partial alignments of 2,562 nuclear genes without evidence of positive selection calibrated on fossil data.** The Bayes Factor test shows no difference with the clock-enforced tree and clock-non-enforced tree. Blue 95% HPD node bars are filled according to posterior probability. Vertical arrows mark the approximate timing of the opening of the Bering Strait [69]. The scale bars denote time based on two dates of calibration based on the fossil record: 13–19 Ma at node A with *12S* mitochondrial sequence (Lee, 2003 rate estimate’) [35] and 5–12 Ma at node C (‘Fossil’) [67].

The strict enforced-clock (marginal model lnL = −905662.53) was not significantly different from a non-enforced-clock (marginal model lnL = −904453.79) for the N4N dataset (Bayes Factor *K* = 0.99867). A strict clock-enforced tree calibrated to the estimated divergence between members of clade S sharing an LCA with *S. purpuratus* based on fossil records show a rapid divergence of clade S in a period of 3–5.5 Ma (Figure 4). The strict clock-enforced tree calibrated to the estimated divergence between members of clade S and clade M based on *12S* mitochondrial genes [35] generated a congruent topology (not shown). The estimated divergence times for each node of the topology from the trees obtained from the two calibration times used in this study are collected in Table 3.

**Table 3:** Divergence time estimates

**Internal node**	**Fossil estimate**	***12S *rDNA rate estimate**	***12S *rDNA Lee (2003)**
A	6.2-11.2	13.3-19.0	13-18
B	4.5-8.2	9.74-14.0	7.2-10
C	3.1-5.6	6.6-9.4	4.6-6.6
D	2.8-5.1	6.0-8.6	
E	2.5-4.5	5.3-7.6	2.1-3.1
F	2.0-3.6	4.2-6.1	
G	3.4-6.1	7.1-10.3	
H	2.7-4.8	5.6-8.1	5.7-8.1

The divergence time estimates for internal nodes of the phylogeny of Strongylocentrotidae based on strict clock estimates calibrated to fossil record and previous studies. Labels for the internal nodes match those in Figure 4. A calibration of 5–12 Ma. at node C was used for ‘Fossil estimate’ and of 12–19 Ma. at node A was used for ‘*12S* rDNA rate estimate’.

## Discussion

Numerous processes, including horizontal gene transfer (HGT), gene duplication, introgressive hybridization, incomplete lineage sorting and natural selection may all contribute to gene tree histories that do not represent the true species tree [42], resulting in gene trees that do not necessarily reflect species trees [50]., In this group of sea urchins, introgression has been documented between some taxa [37], and of the primary mechanisms of HGT, the possibility of HGT by viral transfer exists but is likely to be extremely rare (G. H. Pogson, personal communication). Despite these factors, integrating information from large numbers of independent loci offers considerable promise to generate robust phylogenies in situations where small number of loci failed to do so [46], although care must be taken to assess the robustness of results in the proper biological context [54]. The two multi-locus molecular phylogenies previously published for Strongylocentrotidae provided strong support for the composition of the major groups, but were unable to resolve the relationships of the species [34,35].

The variation in the evolutionary histories of multiple independent genes are typically addressed with either data partitions with different nucleotide substitution models, or with mixture models allowing for random variation between sites [55]. Recent phylogenomic work has demonstrated the potential poor performance of standard phylogenetic methods due to among-site rate variation, causing shifts in the phylogenetic positions of terminal taxa in well-supported trees generated from different models of nucleotide substitution or by different methods [56]. Our analyses evaluated both the gene level support-based evidence and a concatenated site approach including the implicit model of nucleotide evolution in MP, an explicit model of GTR + I + G with BI and a mixture model allowing for rate variation among sites under ML. Our results did not find discordance between the topologies inferred between methods, or with the nodal support based on the different usage of nucleotide substitution models between the ML, BI and MP analysis of nuclear fourfold degenerate sites of genes without evidence of positive selection. We take the complete concordance between such disparate methods and the morphological data as strong support for the biological significance of these proposed species relationships.

Mitochondrial genes offer potential utility as molecular markers for reconstructing phylogenetic relationships, as the order and number of mitochondrial genes are typically conserved over large phylogenetic distances and orthology is clear [57]. However, mitochondrial phylogenies may be misleading [58], a fact we find in our data best represented by the incongruence and limited node support between BI, MP and MP methods with the concatenated mitochondrial MA and M4 data. Our results using mitochondrial genes, and those of previous studies in this group, produce conflicting topologies and do not demonstrate clear or consistent signals of phylogenetic relationships.

### Strongylocentrotus and Mesocentrotus

For this study, we have chosen to follow the taxon details of the World Echinoidea Database [28] and acknowledge the *Mesocentrotus* genus and identify *S. fragilis* (nee *Allocentrotus fragilis*). Indeed, the molecular evidence from this study strongly support the membership of *M. franciscanus* and *M. nudus* to a group sister to *Strongylocentrotus*. Our results confirm the two major clades of *Strongylocentrotus* and *Mesocentrotus* previously identified by mitochondrial gene studies [34,35]. Clade S forms a monophyletic *Strongylocentrotus* and *Hemicentrotus* group supporting the inclusion of *S. fragilis*. Clade M conforms to the proposed *Mesocentrotus* group [33], including *M. franciscanus*, *M. nudus* and *P. depressus*. The molecular distinction between *Strongylocentrotus* and *Mesocentrotus* taxa is also supported by recent morphological classifications of the cross-section of the ultrastructure of primary spines [59].

Previous studies suggested *H. pulcherrimus* was an early branching member of clade S [34,35]. Our data support *H. pulcherrimus* as an early branching member of this clade [35], rather than sister to *S. intermedius*[34].

### Divergence patterns and speciation

Population disjunctions, such as vicariant events and limitations to dispersal, are important first steps towards allopatric speciation [60]. Vicariant events due to sea level changes are well documented across the Isthmus of Panama [61], Baja California [62] and the Bering Strait [63]. Sea levels experienced a severe decline at 10.5 Ma with regular fluctuations occurring since [64]. This fluctuation broadly corresponds to the “Vicariant Pacific Pattern” (VPP), where amphi-Pacific taxa gave rise to eastern and western Pacific forms [65] during the Neogene.

Parsimoniously, our phylogeny suggests a western Pacific (WP) last common ancestor living in shallow, warmer waters followed by an expansion into the WP ancestor of the two major clades. Descendants of each clade experienced two separate eastern Pacific (EP) invasions (*S. purpuratus* and *M. franciscanus*). In Clade S, a single deep, cold-water invasion at the ancestor of *S. fragilis* and *S. pallidus* occurred, with the LCA of *S. pallidus, S. droebachiensis* and *S. intermedius* invading the Arctic and becoming holarctic (HA) in range. Surprisingly, our data provide strong support for a sister grouping of *S. droebachiensis* and *S. intermedius*. This suggests that *S. intermedius* has invaded the WP and moved into shallower and warmer water.

The sister species of *M. nudus* and *M franciscanus* show disjunct distributions, with one species inhabiting the northwest and the other the northeast Pacific, respectively. The estimated divergence time between these two species of 2.7-4.8 Ma using the fossil record calibration is more recent than the 5.7-8.1 Ma estimated from *12S* mitochondrial DNA but still corresponds with the sea level fluctuations and fit with the VPP [35]. This estimated time of divergence also corresponds to the split between *P. depressus* and the ancestor of *M. nudus* and *M. franciscanus,* suggesting a corresponding event occurring the northern Pacific. In the other sister pair, *S. droebachiensis* inhabits the holarctic region and overlaps the distribution of it’s sister, *S. intermedius*. Extant sister species, however, may not be true sisters as other lineages may be extinct. In addition, current ranges many not reflect historical ones. It is not clear from this phylogeny as to whether these two species likely diverged through allopatric or sympatric means [15]. Interestingly, this habitat overlap becomes marginal if *S. pulchellus* is a distinct species, and not a synonym of *S. droebachiensis*. Major morphological work on the group found *S. pulchellus* agrees with *S. intermedius* in all morphological structures examined except for the tooth skeleton [66]. Future molecular and morphological work will certainly shed light on this divergence.

The members of Clade S show rapid evolutionary divergence along with habitat expansions and changes following a split from a WP ancestor, conforming to the VPP. Isolated spines fossil evidence places undefined *Strongylocentrotus* in the northeast Pacific in the late Miocene [67] though the reliability of these identifications remain suspect [68]. The opening of the Bering Strait would provide the access into arctic habitats necessary for a holarctic expansion [34,35]. The Bering Strait opened at the end of the Miocene, 5.32 Ma [69], overlapping the early bounds of our estimated divergence time of 3.1-5.6 Ma from fossil calibration for the clade containing *S. purpuratus, S. fragilis, S. pallidus, S. droebachiensis* and *S. intermedius*. Furthermore, distinct *S. purpuratus, S. droebachiensis,* and *M. franciscanus* fossils are seen in California formations of the middle Pliocene and *S. droebachiensis* fossils reached western Europe by the late Pliocene [67], supporting a late Miocene, early Pliocene divergence.

The Strongylocentrotidae has two deep-water species, *S. fragilis* and *S. pallidus*. Our inferred phylogeny provides evidence for a single radiation into the deep-water habitat. *S. pallidus* is typically found in lower depths [70]. *S. droebachiensis* is also know to reach depths of 1150 m, but is typically found in the shallow sub tidal zone from 0 to 50 m [26,70]. These species coexist in the same geographic range with *S. droebachiensis* in the shallow and *S. pallidus* in the deep habitats. Our tree suggests that *S. pallidus* and *S. fragilis* share a recent common ancestor from a single deep-water invasion and as such may share adaptations to this environment. Indeed, adaptations for the deep-water habitat invasions of *S. fragilis* have been proposed based on genome-wide comparative analysis of three species (not including *S. pallidus* or *S. droebachiensis*) [12]. However, gamete production declines with depth, and the very deep-water individuals of *S. fragilis* aren’t expected to be spawning (John Pearse, personal communication). If that is the case, then natural selection may not reach the very deep-water habitats and deep-water adaptations would be based on selection pressures found at the more shallow depths. With these genome-wide comparative data, future research can test for molecular adaptations along the branch leading to the ancestor of these taxa as well identify adaptations unique to the branches leading to these extant taxa.

Vicariance is insufficient to completely explain our observed pattern of divergence between these taxa, and much work has been done in this group to explore the effects of rapidly evolving gamete recognition proteins on isolating populations [14,36,39,71]. However, the putative egg receptor protein, EBR1, for the sperm bindin gamete recognition protein in sea urchins is prohibitively long for traditional sequencing methods [13]. The phylogenetic relationships inferred from our extended genomic sampling offer a unique opportunity to expand hypothesis of molecular evolution and adaptation in this group of sea urchins.

## Conclusions

This phylogeny was consistent with a single invasion of deep-water environments followed by a holarctic expansion by *Strongylocentrotus*. Divergence times for each species estimated with reference to the divergence times between the two major clades of the group suggest a correspondence in the timing with the opening of the Bering Strait and the invasion of the holarctic regions. However, vicariance is insufficient to completely explain the divergence between these taxa and other isolating mechanisms are appropriate to explore in this system. In particular, much work has been done to explore the effects of rapidly evolving gamete recognition proteins on isolating populations in sea urchins. The phylogenetic relationships inferred in this study and the comparative genomic data now available provide a unique opportunity to explore hypothesis of molecular evolution and adaptation in natural populations.

## Methods

### Mitochondrial genome nucleotide sequences

Class Echinoidea has been found to be monophyletic in Echinoderm phylogenies inferred from both mitochondrial DNA (mtDNA) sequences and morphological data [29-31]. The family Strongylocentrotidae consistently resolves as sister to *Paracentrotus lividus*, *Echinocardium cordatum* and *Arbacia lixula* in both molecular and morphological phylogenies [31,72].

The complete mitochondrial genomes available for six urchin species were obtained from GenBank (*Strongylocentrotus droebachensis* NC 009940; *Strongylocentrotus purpuratus*, NC 001453; *Strongylocentrotus pallidus*, NC 009941; *Paracentrotus lividus*, NC 001572; *Arbacia lixula*, NC 001770; *Echinocardium cordatum*, FN562581.1).

The published sequences for all nine strongylocentrotid species were collected for regions covering *COI, COII, tRNA-Lys, ATPase8 and ATPase6* [GenBank:AY220998-AY221021] [34]. The published sequences for *S. intermedius*, *S. nudus* and *H. pulcherrimus* were collected for *COI* [GenBank:AF525455, GenBank:AF525452 and GenBank:AF525453, respectively], *NDI* [GenBank:AF525454, GenBank:AF525450 and GenBank:AF525451, respectively] and *12S* [GenBank:AF525769, GenBank:AF525767 and GenBank:AF525768, respectively) [35].

We supplemented the mitochondrial genomes collected from GenBank with *de novo* assemblies from Illumina paired-end reads of genomic. Reference sequences for twelve protein-coding (*COI, COII, ND1, ND2, ND3, ND4, ND4L, ND5, ND6, CytB, ATPase6* and *ATPase8*) and two ribosomal RNA (*12S* and *16S*) genes were identified in each mitochondrial genome based on the annotated nucleotide sequence from *S. purpuratus* [GenBank:NC001543].

### Mitochondrial genome de novo assemblies and annotation

Mitochondrial genomic sequences were assembled *de novo* from Illumina paired-end reads of genomic DNA (Kober and Pogson, unpublished data). First, to obtain a set of putative mitochondrial DNA reads, all reads for each species were aligned to all six GenBank mitochondrial genome sequence with SSAHA2 [73] using parameters ‘-solexa -skip 6 -pair 20,3000’. All reads that mapped to any of the six molecules with a mapping quality of greater than 5 were collected for each species.

The collected reads were used as input for *de novo* assembly of the molecule for each species using velvet [74]. Hash size values between 11 and 99 were evaluated using VelvetOptimiser.pl [75] and optimized for the total number of base pairs in large contigs. For *S. fragilis*, previously sequenced 454 reads [12] were also aligned to *S. purpuratus* [GenBank:NC 001453] with SSAHA2. 454 reads aligning with a mapping quality >5 were also included with the Illumina paired end reads as input to the *S. fragilis de novo* assembly. Our assembled *M. franciscanus* molecule was included as an additional reference sequence for obtaining putative mitochondrial reads for the *de novo* assembly for *M. nudus*, *P. depressus* and *H. pulcherrimus*. *De novo* contigs over 1000 bp for *P. depressus* were collected and the partial mitochondrial genome was assembled from two non-overlapping contigs generated with CAP3 [76]. These assembled mitochondrial genomes then provided the template from which we identified and extracted the nucleotide sequence of each gene for each species.

Reference sequences for twelve protein-coding (*COI, COII, ND1, ND2, ND3, ND4, ND4L, ND5, ND6, CytB, ATPase6* and *ATPase8*) and two ribosomal RNA (*12S* and *16S*) genes were identified in each mitochondrial genome based on the annotated nucleotide sequence from *S. purpuratus* [GenBank: NC 001543]. We identified the start and stop coordinates for each gene location on the *de novo* assembled mitochondrial genomes for each species by aligning the *S. purpuratus* gene reference sequence to our *de novo* assembled mitochondrial genome for each species using BLAT [77] with DNA sequences translated in six frames to protein and allowing one mismatch in the tile.

The sequence of protein coding mitochondrial genes we identified were aligned using transAlign [78] using the echinoderm mitochondrial code. The mitochondrial ribosomal RNA (rRNA) *12S* and *16S* gene sequences were aligned using clustalw2 [79]. Ambiguously aligned regions were identified and removed with Gblocks [80] with default parameters and no gap positions allowed. Two sequences for each gene were obtained for *S. droebachiensis* and *S. pallidus*: one from the GenBank mitochondrial genome sequence and the other from our *de novo* assembly. The *de novo* assembled mitochondrial genomes and the predicted gene models were submitted to GenBank [GenBank: KC898196-KC898203].

### Nuclear genome nucleotide sequences

Briefly, we defined nuclear genes based on the gene model coordinates defined in SpBase Build 6 based on the Spur v3.1 genome assembly (SpBase.org). The alignments generated from the genomic reads of *S. purpuratus* were used to represent that species, rather than the reference genome sequence. We discarded any gene model that was not manually annotated, incomplete (i.e. no internal stop codons, missing valid start or stop codon) or were putative in-paralogs (i.e. annotated as paralogs or of overlapping coordinates). Partial alignments of nuclear genes including ambiguous sites (i.e. heterozygote) were constructed from alignments of Illumina paired-end reads of nine species (Kober and Pogson, unpublished data) aligned to the *S. purpuratus* (Table 1). Paired-end short reads were aligned to Spur v3.1 using SSAHA2. Reads with a mapping quality of <30 were discarded. Nucleotides with a quality score of <25 were ignored. Heterozygotes sites were called when more than one allele was represented by a frequency of >0.20 and >10 valid nucleotides were present from aligned reads. We excluded alignments of greater than 100 unambiguous codons across all nine taxa, leaving 3,180 for analysis.

Additional tools used in the analyses included James Kent’s source tools [81], Biopython (http://biopython.org), FigTree (http://tree.bio.ed.ac.uk/software/figtree/), R [82], bedtools [83], EMBOSS [84] and the Newick utilites [85].

### Concatenated alignments and tests for positive Darwinian selection

We created a concatenated alignment from the Gblocks masked alignments of mitochondrial genes (“MA”) and a concatenated alignment of mitochondrial genes from fourfold degenerate sites (“M4”) identified by codeml from PAML version 4.5 [86]. Fourfold degenerate sites are identified in codeml as third position sites of a codon which are synonymous across all taxa in the alignment (Ziheng Yang, personal communication). Inference of positive Darwinian selection on mitochondrial protein coding genes was performed with codeml from the PAML package [86]. The M7 and M8 models were used in an LRT test and significance was assessed based on a chi-square distribution with two degrees of freedom.

We identified nuclear genes with alignments of greater than 100 unambiguous codons across all nine taxa. A signal of positive Darwinian selection for a gene was defined as having a *q-value* < 0.05 based on the likelihood ratio test between models M7 and M8 implemented in codeml as described above for the mtDNA alignments. The most likely ML tree for these genes was used to represent the inferred phylogeny of that gene. We created concatenated alignments of 4-fold degenerate sites identified by codeml for all nuclear genes (“N4A”), nuclear genes with evidence for positive Darwinian selection (“N4S”) and those without any signal of positive selection (“N4N”).

The alignment of mitochondrial gene sequences newly obtained in the present study and the concatenated alignments of the fourfold degenerate sites of nuclear genes have been deposited in TreeBase (http://purl.org/phylo/treebase/phylows/study/TB2:S13990 ).

### Phylogenetic reconstructions

We used two main approaches to reconstruct the phylogeny of the group using mitochondrial and nuclear genes. The first was a support-based method, which evaluated the individual trees generated for each gene in the nuclear or mitochondrial genome. For the mitochondrial genes, this included an additional analysis accounting for pairing in the RNA secondary structure. The second main approach used the concatenation of sites between all genes in the nuclear or mitochondrial genome, respectively.

The ML tree for each mitochondrial gene was generated using PhyML with the best-fitting nucleotide substitution model, optimized tree topology, branch length and rate parameters, the best tree topology of NNI and SPR search operations, and 10 bootstrap replicates. The best fitting nucleotide substitution model was identified for each mitochondrial gene based on the AICc criterion evaluating 56 models using pmraic version 1.1 (http://www.abc.se/~nylander/mraic/pmraic.html) and PhyML 3.0 v. 20110919 [87].

For *12S* and *16S*, a Bayesian Inference (BI) partitioned analysis of RNA paired stem and unpaired loop sites [88] in were performed using PHASE 2.0 [89]. We predicted a consensus secondary structure from each alignment using RNAalifold [90]. Unpaired regions were analyzed under the general time-reversal REV [91] and paired stem regions were analyzed under the time reversible seven state RNA7D [92,93]. We used a discrete-gamma model with six categories to approximate the Γ-distribution with no invariant sites allowed. We performed 1.5 million sampling iterations with a sampling period of 150 and burn-in iterations of 750,000. The remaining parameters followed Hudelot and colleagues [94].

For each nuclear gene consensus alignment, an ML tree was generated using PhyML using the estimated rate and probability of each class from the data (‘free_rates’), optimized tree topologies, branch length and rate parameters, the best tree topology of NNI and SPR search operations, and 100 bootstrap replicates.

For the concatenated ‘MA, ‘M4’, ‘N4A’, ‘N4S’ and ‘N4N’ alignments, we performed phylogenetic reconstructions using Maximum Parsimony (MP), Maximum Likelihood (ML) and Bayesian Inference (BI) methods in a uniform fashion. The ML analyses of concatenated datasets were performed with PhyML using the estimated rate and probability of each class from the data (‘free_rates’), optimized tree topologies, branch length and rate parameters, the best tree topology of NNI and SPR search operations, and 100 bootstrap replicates. The MP analyses of concatenated datasets were conducted using PAUP* version 4b10 [95] and consisted of heuristic searches with 100,000 replicates of random stepwise addition and TBR branch swapping. Bootstrapping was done using 500,000 ‘fast-bootstrap’ pseudo-replicates. The BI analyses of concatenated datasets were performed using MrBayes v. 3.2.1 [96] assuming a nucleotide substitution model with a gamma-distributed rate variation across sites and a proportion of sites invariable (GTR + I + G). Each dataset was run with four Markov chains for one million generations and sampled every 100 generations. Each analysis was run four times. The first 2500 trees from each run were discarded so that the final consensus tree was based on the combination of accepted trees from each run (a total of 30,004 trees). We tested the convergence between the four runs by examining the potential scale reduction factors (PSRF) produced by the ‘sump’ command in MrBayes. Support for nodes was determined using posterior probabilities (PP, calculated by MrBayes).

### Assessment of significance of differences between trees

To determine if there were significant differences between two proposed trees given the data, we performed the SH test [97] using RELL bootstrap with 1000 replicates [98] and the HKY85 model of nucleotide substitution in PAUP*. We ascribed significance to a *P-value* < 0.05 as provided by the output.

### Molecular clock and divergence times

A strict molecular-clock was tested against a non-clock model assuming a nucleotide substitution model with gamma-distributed rate variation across sites and a proportion of sites invariable (GTR + I + G) using MrBayes. Each dataset was run with four Markov chains for 500,000 generations to confirm PP convergence. The harmonic means of the likelihoods of the MCMC sampling were used as the marginal model likelihoods. A ratio exceeding 5 was considered very strong evidence favoring one model over the other [99]. A strict clock-enforced BI tree with uniform branch lengths was used to estimate the divergence time of each species with MrBayes. We estimated a rate of substitutions per site per million years of 0.01 ± 0.005 and an exponential distribution with a rate of 0.1 for the tree age prior. One topology was tested with two divergence times. One calibration had a divergence time of 5–12 Ma for the ancestor *S. intermedius* and *S. droebachiensis* (Figure 4) based on the fossil record appearance of *S. droebachiensis* in the mid-Pliocene and identifiable *Strongylocentrotus* in the late Miocene [67]. A second calibration used a divergence time between the (*Strongylocentrotus*, *Hemicentrotus*) clades and the (*Paracentrotus, Mesocentrotus*) clades of 13–19 Ma from *12S* mitochondrial genes calibrated using a reference point estimated from the fossil record [35]. Both of these calibrations remain within the Echinidae–Strongylocentrotidae divergence tentatively estimated to be at 25 Ma. [100].

## Competing interests

The authors declare that they have no competing interests.

## Authors’ contributions

KMK and GB conceived the ideas for the study and designed the analysis. KMK carried out the data collection and analysis. KMK and GB interpreted results. GB provided essential biological materials. KMK and GB wrote the manuscript. Both authors read and approved the final manuscript.

## Supplementary Material

Additional file 1: Figure S1 The density tree of the most likely trees obtained from the Maximum Likelihood analysis of putatively neutral nuclear genes (See text for details). Click here for file

Additional file 2: Figure S2 Most likely ML tree for NADH dehydrogenase subunit mitochondrial genes. Node support from 10 bootstrap replicates. Click here for file

Additional file 3: Figure S3 Most likely ML tree for protein coding mitochondrial genes. Node support from 10 bootstrap replicates. Click here for file

Additional file 4: Figure S4 Most likely ML tree for ribosomal RNA mitochondrial genes. Node support from 10 bootstrap replicates. Click here for file

Additional file 5: Figure S5 Cladograms produced from *12S* sequences. Sequences (A) as presented in Fig. 2 of Lee (2003) and (B) ML methods with sequences of Lee (2003) and additional sequences used in this study. Branches are labeled with ML bootstrap values. Click here for file
