# Erratum to: Phylogenomics of strongylocentrotid sea urchins

**DOI:** 10.1186/s12862-017-0875-5

**Published:** 2017-02-13

**Authors:** Kord M. Kober, Giacomo Bernardi

**Affiliations:** 1 Department of Ecology & Evolutionary Biology, University Of California Santa Cruz, Santa Cruz, CA USA; 2 Department of Physiological Nursing, University Of California San Francisco, San Francisco, CA USA

## Erratum

The original version of this manuscript [1] unfortunately contained a mistake. Two species labels mentioned throughout the main body and supplementary files were swapped. The samples have since been re-verified by Sanger sequencing from stock DNA (not shown).

**Fig. 1:**
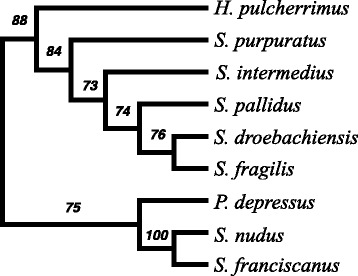
The cladogram of the most frequent tree obtained from the Maximum Likelihood analysis of 2301 nuclear genes without evidence of positive selection. Branch support is quantified as the frequency that the node is supported by a gene alignment where the most frequent tree was not rejected or the gene’s ML tree was significantly different from the most frequent tree (see text). The tree is rooted between the two major clades identified in this group

**Fig. 2:**
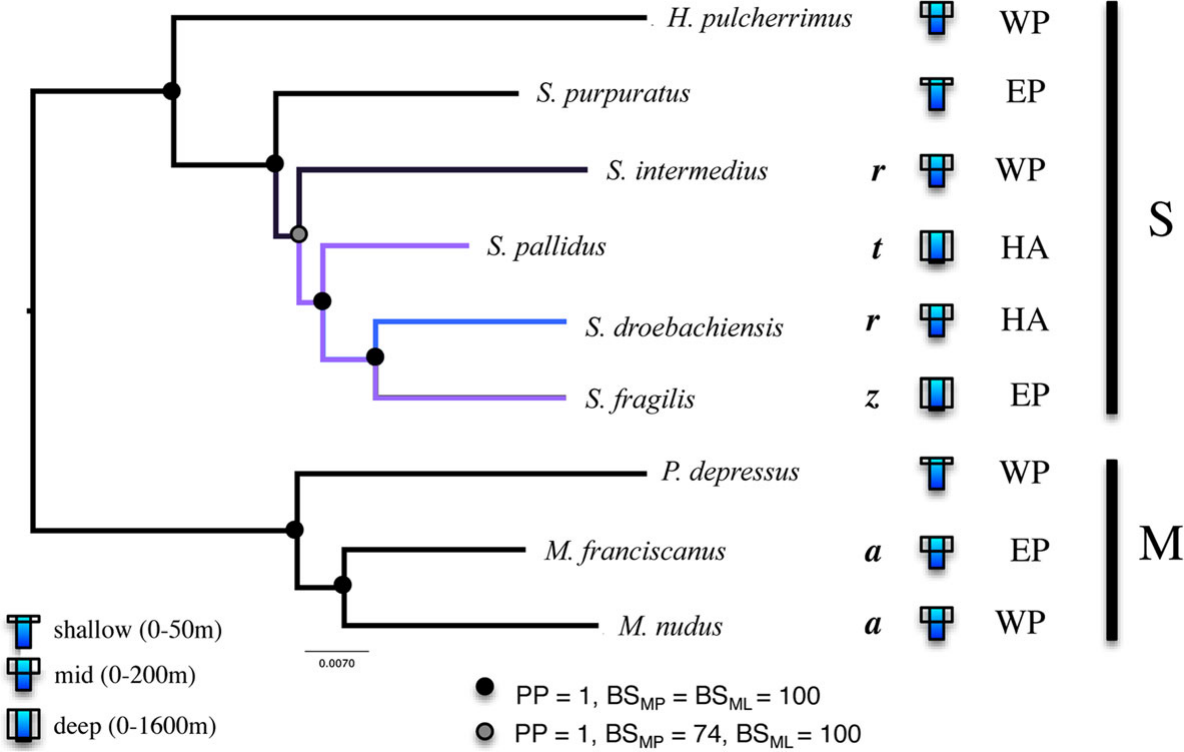
The 50% majority rule consensus phylogram of the stationary trees obtained from the Bayesian inference analysis of concatenated neutral nuclear genes at four-fold degenerate sites mid-point rooted between the two major clades previously identified. Branch support values are the BI posterior probabilities (PP), MP bootstrap (BS_MP_) and ML bootstrap (BS_ML_) for genes rejecting evidence of positive selection. Branches leading to deep water species are colored in purple. The branch leading to *S. droebachiensis* is colored blue, as this species occurs primarily in shallow water but can range to a depth of 300 m. Adult depth range: s, shallow (0-50 m); m, medium (0-200 m); d, deep (0-1600 m). Distributions: West Pacific (WP), East Pacific (EP), holarctic (HA). The cross-section of the ultrastructure of primary spines [59]: rectangular (*r*), trapezoid (*z*), triangular (*t*) or ansiform (*a*)

**Fig. 3:**
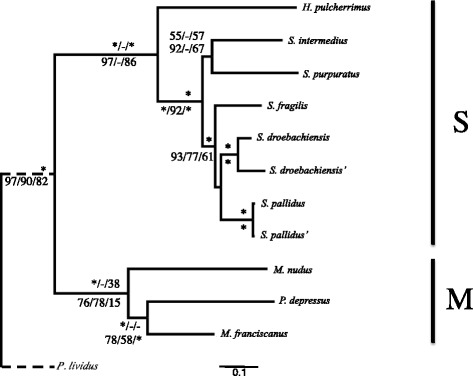
The 50% majority rule consensus phylogram of the stationary trees obtained from the Bayesian inference analysis of concatenated mitochondrial genes at all sites. Branch support are the Bayesian Inference posterior probabilities (BI PP), Maximum Parsimony bootstrap (MP BS) and Maximum Likelihood bootstrap (ML BS) for concatenated mitochondrial genes above and four-fold degenerate sites below the branch. Asterisks on the branch labels denote strong support for the method or all methods (BI PP > =99, MP BS > =95, ML BS > =95). Unsupported nodes are indicated with ‘-‘. Single quotation marks next to a taxon name denote the *de novo* assembled individual from this study of the species. Scale bar, substitutions per site

**Fig. 4:**
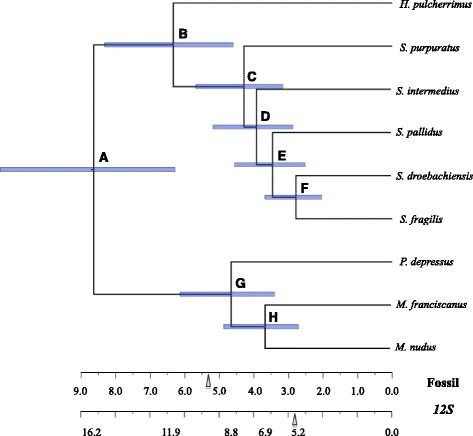
The molecular clock enforced dated phylogram from Bayesian Inference (BI) among fourfold degenerate sites from partial alignments of 2,562 nuclear genes without evidence of positive selection calibrated on fossil data. The Bayes Factor test shows no difference with the clock-enforced tree and clock-non-enforced tree. Blue 95% HPD node bars are filled according to posterior probability. Vertical arrows mark the approximate timing of the opening of the Bering Strait [69]. The scale bars denote time based on two dates of calibration based on the fossil record: 13–19 Ma at node A with *12S* mitochondrial sequence (Lee, 2003 rate estimate’) [35] and 5–12 Ma at node C (‘Fossil’) [67]

Any occurrences of these species names have been corrected in the main text as shown below. In addition, a corrected version of Table 1 is provided below with the updated versions of the figures 1, 2, 3 and 4 and the supplementary files affected.

Page 1. Abstract should read as follows

Proposed text

In contrast, we obtained a very well-supported phylogeny from 2301 nuclear genes without evidence of positive Darwinian selection both from the majority of most-likely gene trees and the concatenated fourfold degenerate sites: ((*P. depressus, (M. nudus, M. franciscanus*), (*H. pulcherrimus,* (*S. purpuratus,* (*S.* ***intermedius*** *,* (*S. pallidus,* (*S. droebachiensis, S.* ***fragilis***)). This phylogeny was consistent with a single invasion of deep-water environments **and** holarctic expansion by *Strongylocentrotus*.

Page 4. Results should read as follows

The BI 50% majority-rule consensus phylogram of the stationary tree inferred from fourfold degenerate sites of nuclear genes without selection (‘N4Ds tree’) had complete (BI PP = 1, BS_ML_ = BS_MP_ = 100) or very strong support from all three methods at all nodes with the N4N and N4A datasets except at the divergence of *S.* ***intermedius*** (BS_MP_ = 74 and BS_MP_ = 69, respectively).

Page 4. Results should read as

Indeed, the tree obtained from the N4S data produced a similar topography except *S. purpuratus* and *S.* ***intermedius*** branching locations were swapped, with *S.* ***intermedius*** the earlier branching of the two with low support for the node (not shown).

Page 6. Results should read as

We also found very strong support for a monophyletic clade of *S.* ***fragilis***, *S. droebachiensis* and *S. pallidus* across our analysis of concatenated datasets. However, MA and M4 datasets produced *S. pallidus* sister to *S. droebachiensis*, but without strong support. In contrast, the N4A, N4S and N4N concatenated datasets found very strong support *S.* ***fragilis*** as sister to *S. pallidus*.

Page 6. Results should read as

We found contradictory topologies for the relative positions of *S.* ***intermedius*** and *S. purpuratus* among mitochondrial genes trees.

Page 6. Results should read as

The locations of *S. purpuratus* and *S.* ***intermedius*** were discordant between the MP method and BI and ML methods in the MA and M4 datasets. BI and ML trees had these two species sister to *S.* ***fragilis***, *S. pallidus* and *S. droebachiensis* (Figure 3), whereas the MP method has *S. purpuratus* branching earliest, then *S.* ***intermedius*** and then the *S.* ***fragilis***, *S. pallidus* and *S. droebachiensis* observed with the nuclear concatenated datasets (not shown). The monophyly of a *S.* ***fragilis***, *S. pallidus* and *S. droebachiensis* clade was recovered in both MA and M4, but we found conflicting support for a sister relationship between *S. pallidus* and *S. droebachiensis* versus *S. pallidus* and *S.* ***fragilis*** (Figure 3).

Page 6. Results should read as

When we included the *12S* sequences of Lee (2003) with our *12S* data, our alignment and ML method produced a **congruent** tree (Additional file 5: Figure S5) and *H. pulcherrimus,* ***S. intermedius*** *,* and *S. nudus* individuals resolved as sister taxa.

Table 1 should also read as follows

**Table:** 

*S. intermedius* (A. Agassiz)	WP	0-225	**15,748**
*S. fragilis* (Jackson)	EP	0-1150	**15,718**

## Additional files

Additional file 1: Figure S1. The density tree of the most likely trees obtained from the Maximum Likelihood analysis of putatively neutral nuclear genes (See text for details). (DOC 530 kb)
Additional file 2: Figure S2. Most likely ML tree for NADH dehydrogenase subunit mitochondrial genes. Node support from 10 bootstrap replicates. (DOC 210 kb)
Additional file 3: Figure S3. Most likely ML tree for protein coding mitochondrial genes. Node support from 10 bootstrap replicates. (DOC 177 kb)
Additional file 4: Figure S4. Most likely ML tree for ribosomal RNA mitochondrial genes. Node support from 10 bootstrap replicates. (DOC 79 kb)
Additional file 5: Figure S5. Cladograms produced from *12S* sequences. Sequences (A) as presented in Fig. 2 of Lee (2003) and (B) ML methods with sequences of Lee (2003) and additional sequences used in this study. Branches are labeled with ML bootstrap values. (DOC 84 kb)
