# Supplementary material for: Phylogenomics of strongylocentrotid sea urchins
Source: BMC Evol Biol. 2013 Apr 23;13:88. doi: 10.1186/1471-2148-13-88 (PMC3637829; doi:10.1186/1471-2148-13-88)

**Additional file 1: Figure S1.** The density tree of the most likely trees obtained from the Maximum Likelihood analysis of putatively neutral nuclear genes (See text for details).


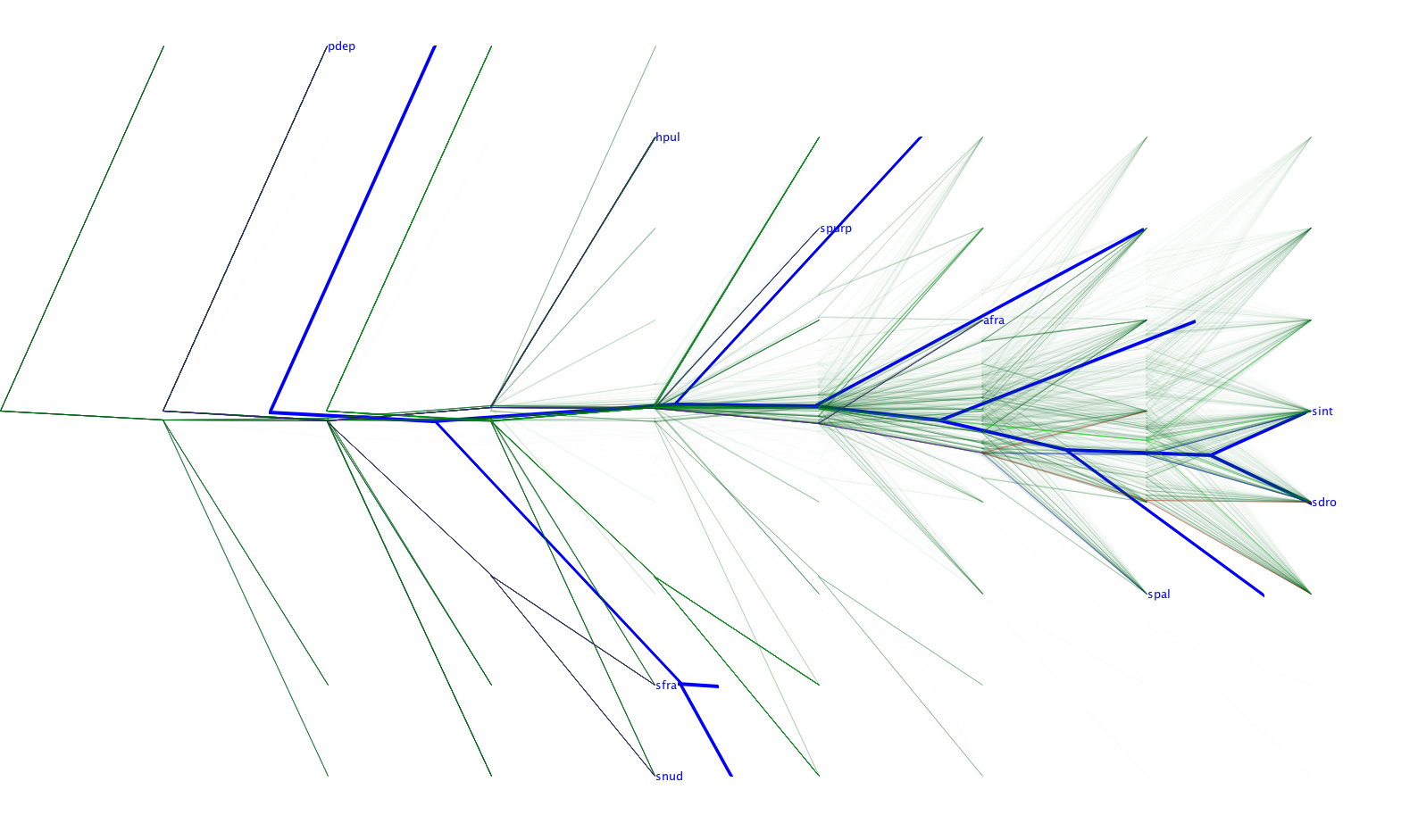

Supplement: Additional file 1: Figure S1 — The density tree of the most likely trees obtained from the Maximum Likelihood analysis of putatively neutral nuclear genes (See text for details). [file 1471-2148-13-88-S1.doc]
