# Supplementary material for: Phylogenomics of strongylocentrotid sea urchins
Source: BMC Evol Biol. 2013 Apr 23;13:88. doi: 10.1186/1471-2148-13-88 (PMC3637829; doi:10.1186/1471-2148-13-88)

**Additional file 2 :Figure S2.** Most likely ML tree for NADH dehydrogenase subunit mitochondrial genes. Node support from 10 bootstrap replicates.

(A) ND1 (B) ND2


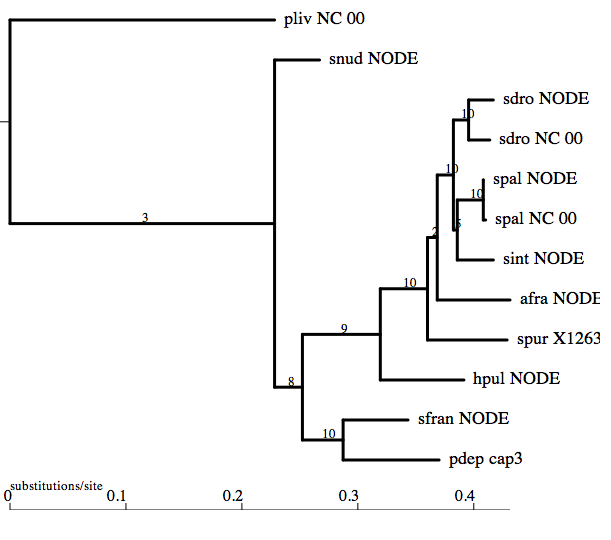
 *
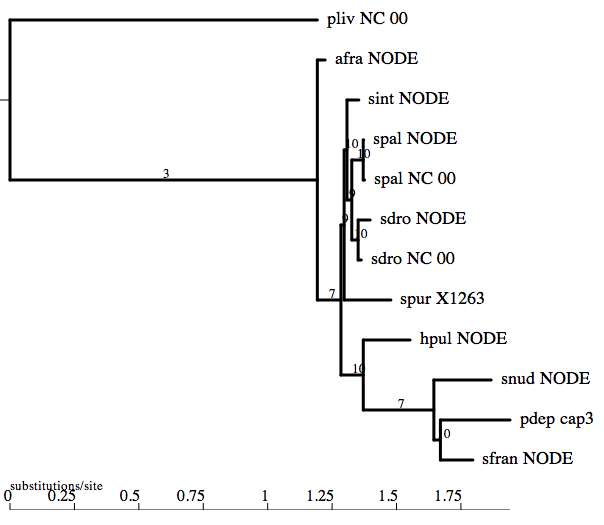
*

(C) ND3 (D) ND4


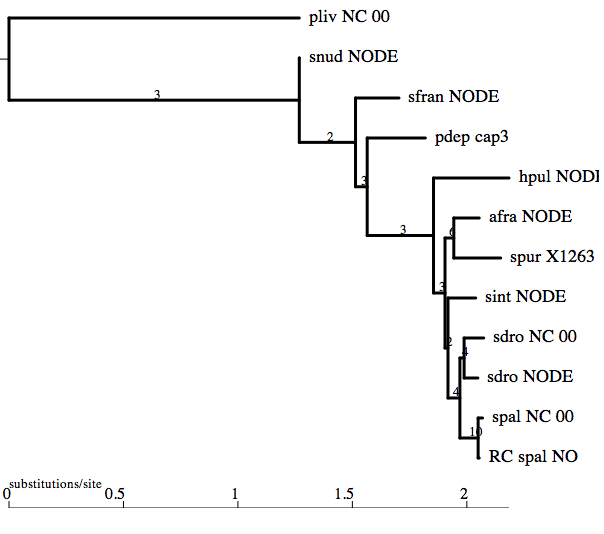

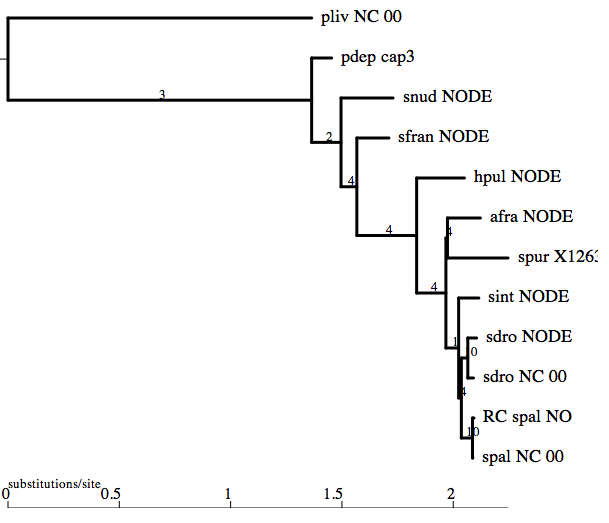


(E) ND4L (F) ND6


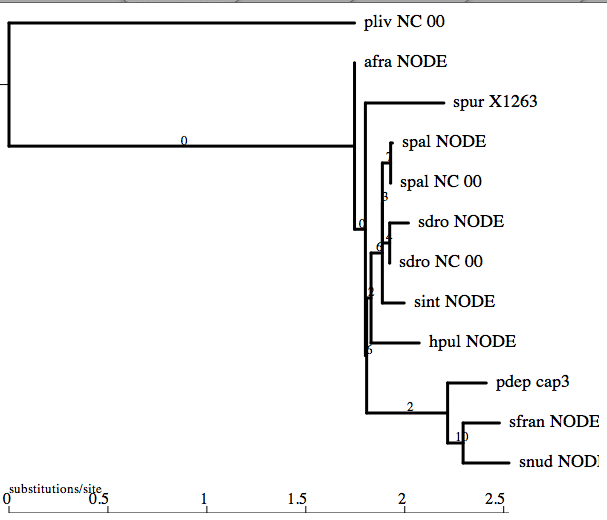

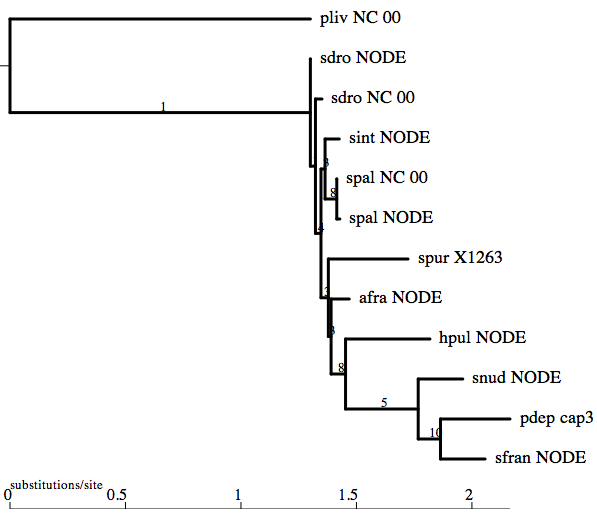

Supplement: Additional file 2: Figure S2 — Most likely ML tree for NADH dehydrogenase subunit mitochondrial genes. Node support from 10 bootstrap replicates. [file 1471-2148-13-88-S2.doc]
