# Supplementary material for: Phylogenomics of strongylocentrotid sea urchins
Source: BMC Evol Biol. 2013 Apr 23;13:88. doi: 10.1186/1471-2148-13-88 (PMC3637829; doi:10.1186/1471-2148-13-88)

**Additional file 3: Figure S3.** Most likely ML tree for protein coding mitochondrial genes. Node support from 10 bootstrap replicates.

(A) COI (B) COII


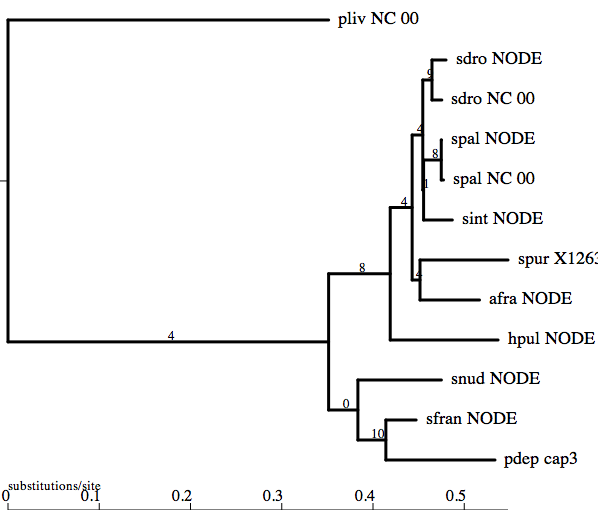

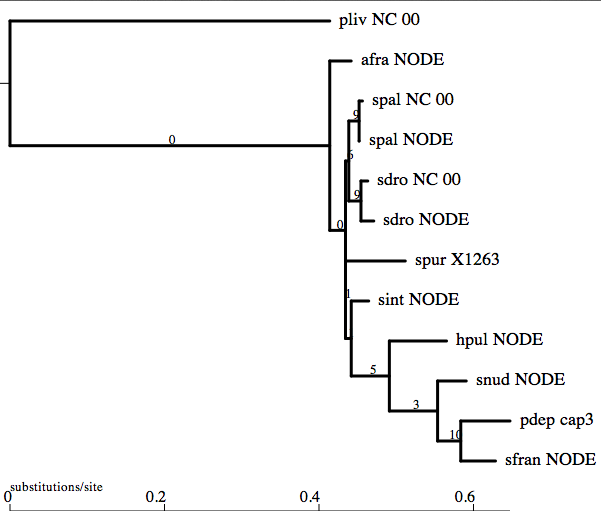


(C) ATPase6 (D) ATPase8


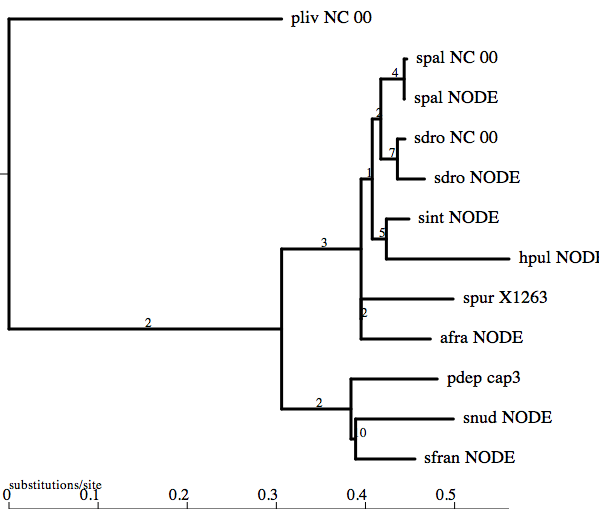

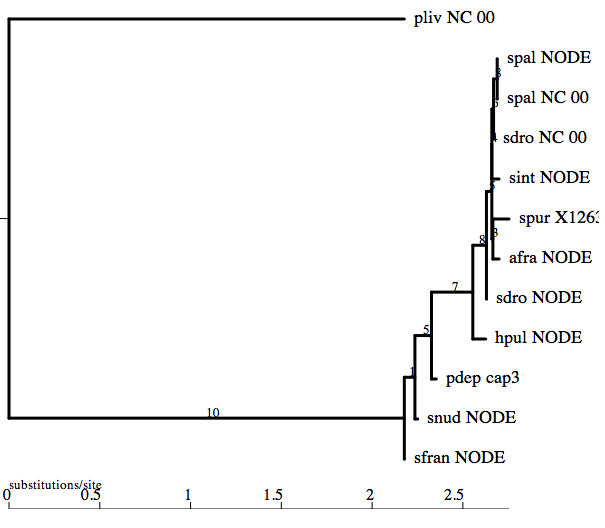


(E) CytB


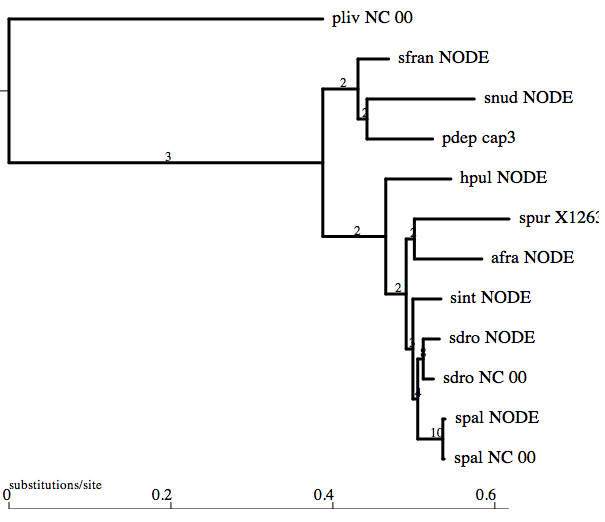

Supplement: Additional file 3: Figure S3 — Most likely ML tree for protein coding mitochondrial genes. Node support from 10 bootstrap replicates. [file 1471-2148-13-88-S3.doc]
