# Supplementary material for: Phylogenomics of strongylocentrotid sea urchins
Source: BMC Evol Biol. 2013 Apr 23;13:88. doi: 10.1186/1471-2148-13-88 (PMC3637829; doi:10.1186/1471-2148-13-88)

**Additional file 4: Figure S4.** Most likely ML tree for ribosomal RNA mitochondrial genes. Node support from 10 bootstrap replicates.

(A) 12S (B) 16S


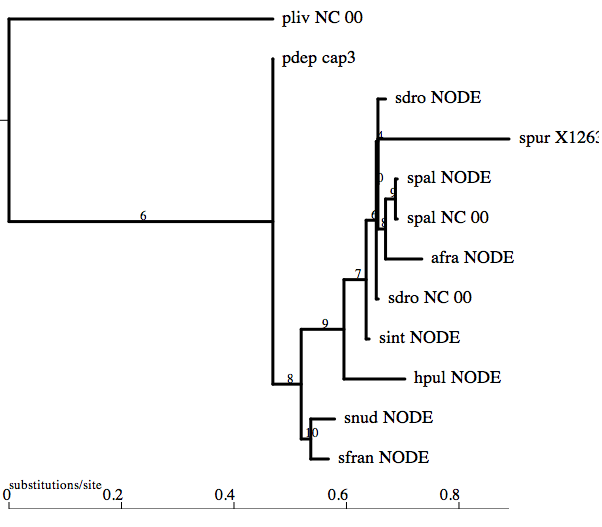

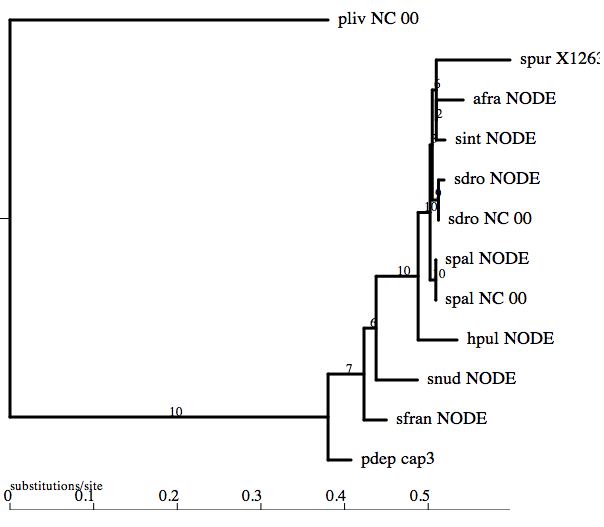

Supplement: Additional file 4: Figure S4 — Most likely ML tree for ribosomal RNA mitochondrial genes. Node support from 10 bootstrap replicates. [file 1471-2148-13-88-S4.doc]
