# Supplementary material for: Phylogenomics of strongylocentrotid sea urchins
Source: BMC Evol Biol. 2013 Apr 23;13:88. doi: 10.1186/1471-2148-13-88 (PMC3637829; doi:10.1186/1471-2148-13-88)

**Additional file 5: Figure S5.** Cladograms produced from *12S* sequences. Sequences (A) as presented in Fig. 2 of Lee (2003) and (B) ML methods with sequences of Lee (2003) and additional sequences used in this study. Branches are labeled with ML bootstrap values.


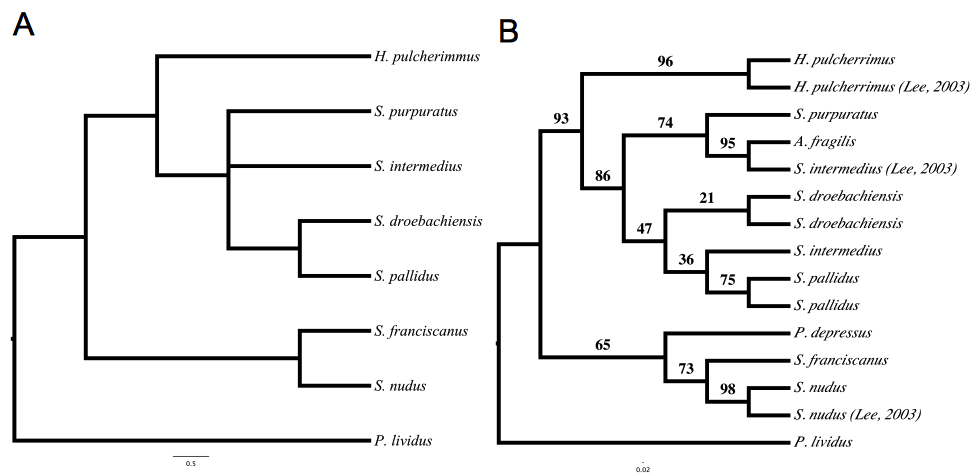

Supplement: Additional file 5: Figure S5 — Cladograms produced from 12S sequences. Sequences (A) as presented in Fig. 2 of Lee (2003) and (B) ML methods with sequences of Lee (2003) and additional sequences used in this study. Branches are labeled with ML bootstrap values. [file 1471-2148-13-88-S5.doc]
