# Supplementary material for: Erratum to: Phylogenomics of strongylocentrotid sea urchins
Source: BMC Evol Biol. 2017 Feb 13;17:50. doi: 10.1186/s12862-017-0875-5 (PMC5307700; doi:10.1186/s12862-017-0875-5)

**Additional file 5: Figure S5.** Cladograms produced from *12S* sequences. Sequences (A) as presented in Fig. 2 of Lee (2003) and (B) ML methods with sequences of Lee (2003) and additional sequences used in this study. Branches are labeled with ML bootstrap values.


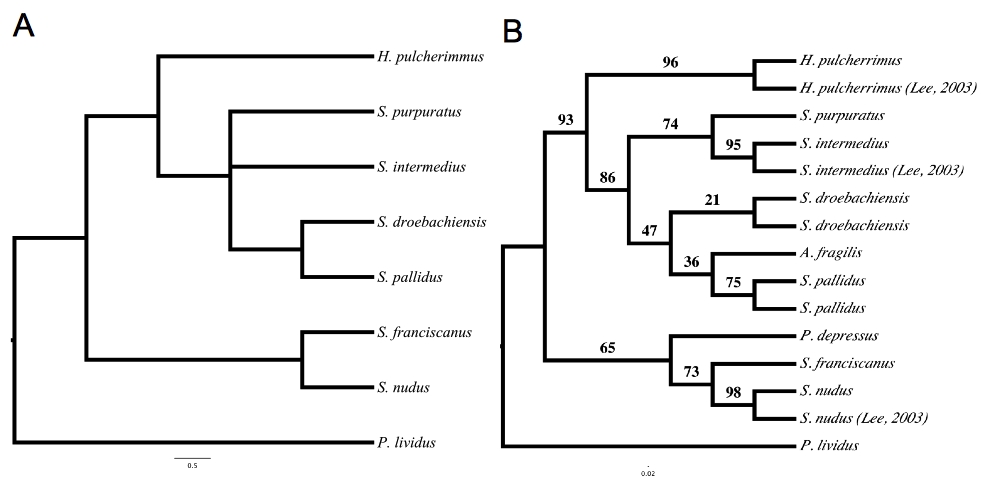

Supplement: Additional file 1: Figure S1. — The density tree of the most likely trees obtained from the Maximum Likelihood analysis of putatively neutral nuclear genes (See text for details). (DOC 530 kb) [file 12862_2017_875_MOESM1_ESM.doc]
