# Supplementary material for: Erratum to: Phylogenomics of strongylocentrotid sea urchins
Source: BMC Evol Biol. 2017 Feb 13;17:50. doi: 10.1186/s12862-017-0875-5 (PMC5307700; doi:10.1186/s12862-017-0875-5)

**Additional file 2 :Figure S2.** Most likely ML tree for NADH dehydrogenase subunit mitochondrial genes. Node support from 10 bootstrap replicates.

(A) ND1 (B) ND2


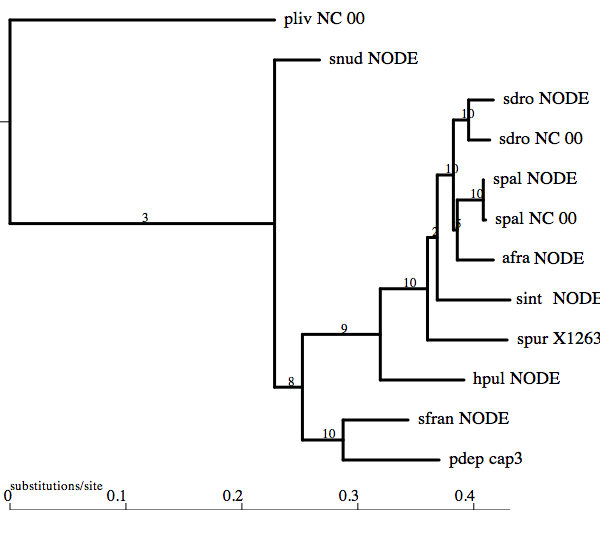

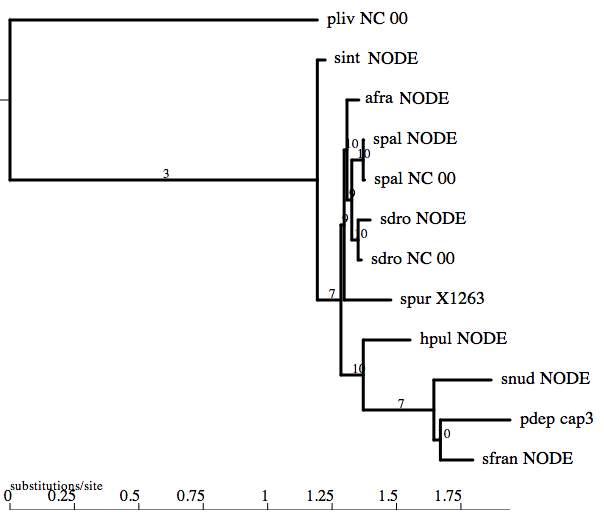


(C) ND3 (D) ND4


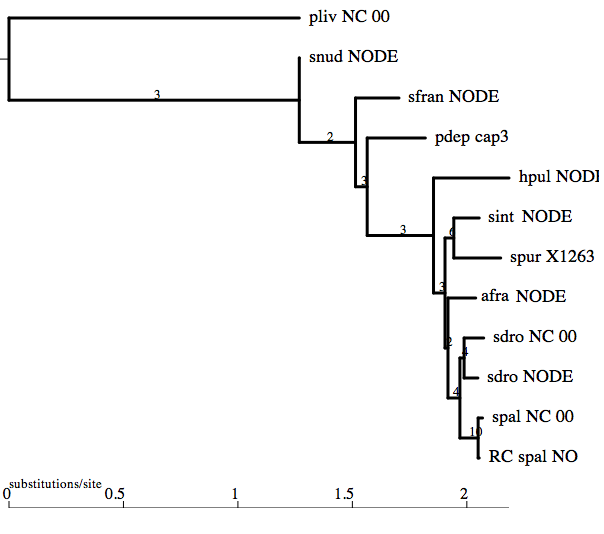

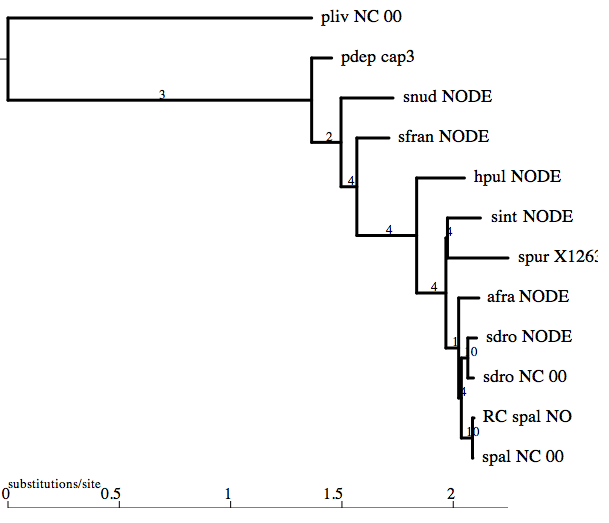


(E) ND4L (F) ND6


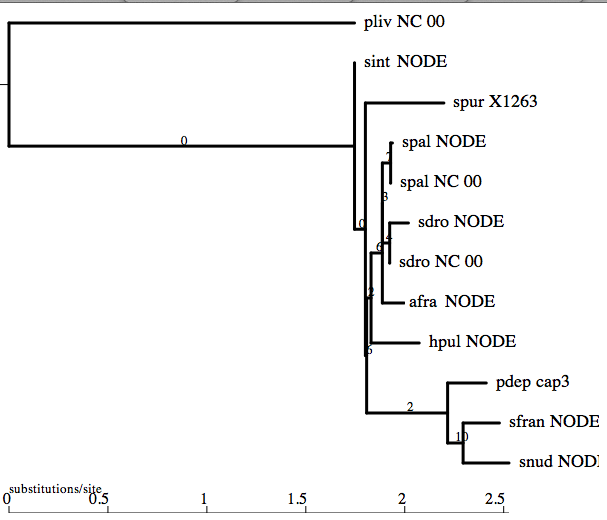

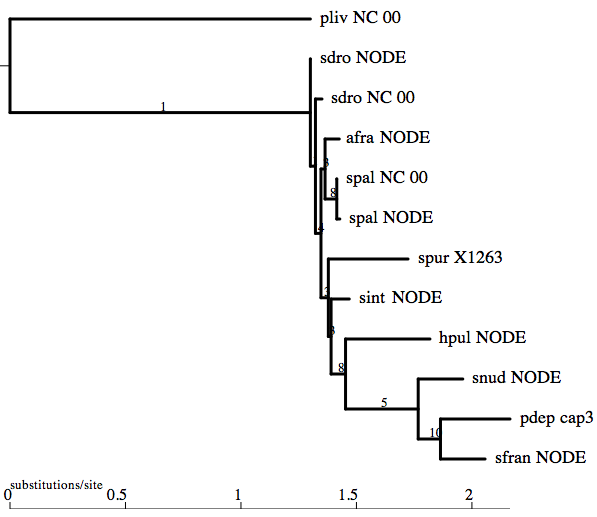

Supplement: Additional file 2: Figure S2. — Most likely ML tree for NADH dehydrogenase subunit mitochondrial genes. Node support from 10 bootstrap replicates. (DOC 210 kb) [file 12862_2017_875_MOESM2_ESM.doc]
