# Supplementary material for: Erratum to: Phylogenomics of strongylocentrotid sea urchins
Source: BMC Evol Biol. 2017 Feb 13;17:50. doi: 10.1186/s12862-017-0875-5 (PMC5307700; doi:10.1186/s12862-017-0875-5)

**Additional file 3: Figure S3.** Most likely ML tree for protein coding mitochondrial genes. Node support from 10 bootstrap replicates.

(A) COI (B) COII


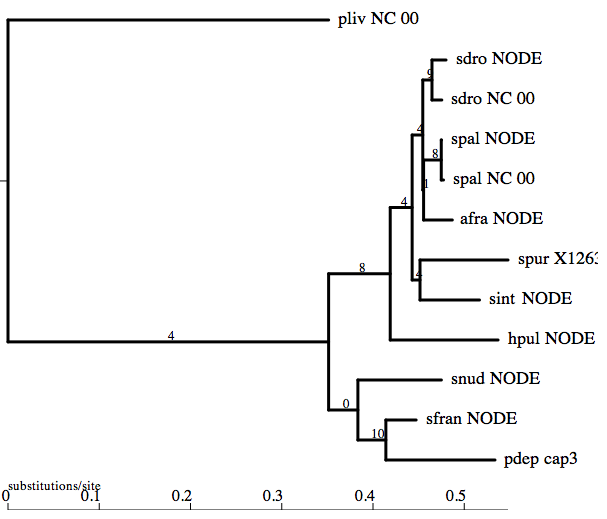

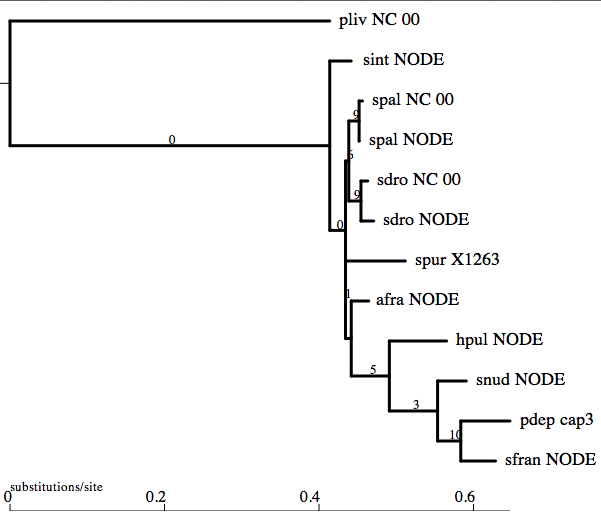


(C) ATPase6 (D) ATPase8


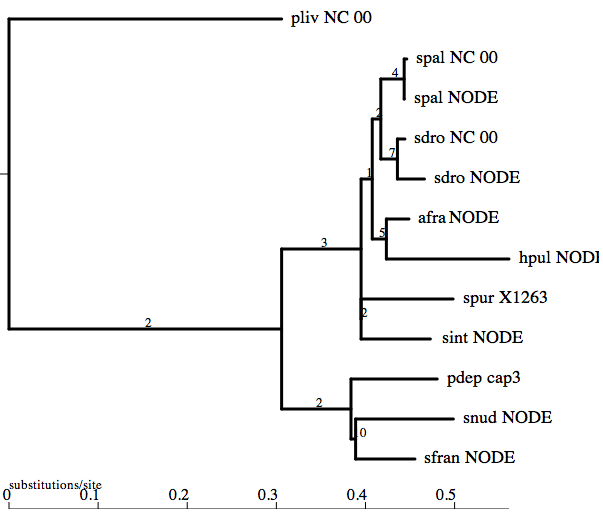

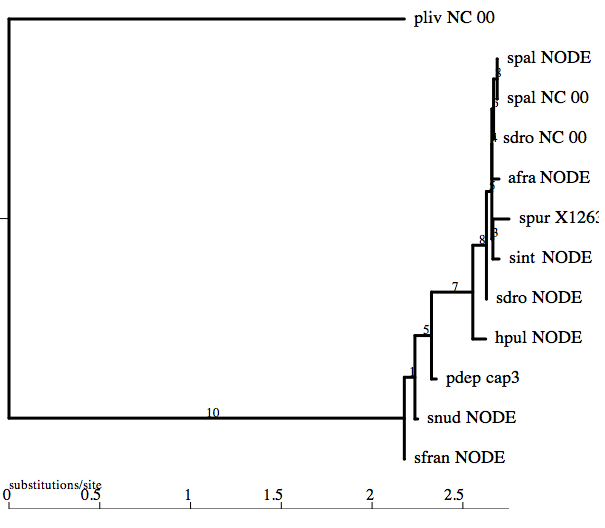


(E) CytB


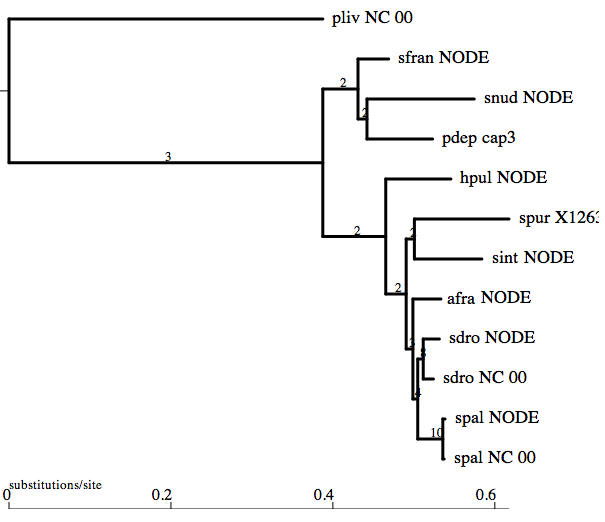

Supplement: Additional file 3: Figure S3. — Most likely ML tree for protein coding mitochondrial genes. Node support from 10 bootstrap replicates. (DOC 177 kb) [file 12862_2017_875_MOESM3_ESM.doc]
