# Supplementary material for: Erratum to: Phylogenomics of strongylocentrotid sea urchins
Source: BMC Evol Biol. 2017 Feb 13;17:50. doi: 10.1186/s12862-017-0875-5 (PMC5307700; doi:10.1186/s12862-017-0875-5)

**Additional file 4: Figure S4.** Most likely ML tree for ribosomal RNA mitochondrial genes. Node support from 10 bootstrap replicates.

(A) 12S (B) 16S


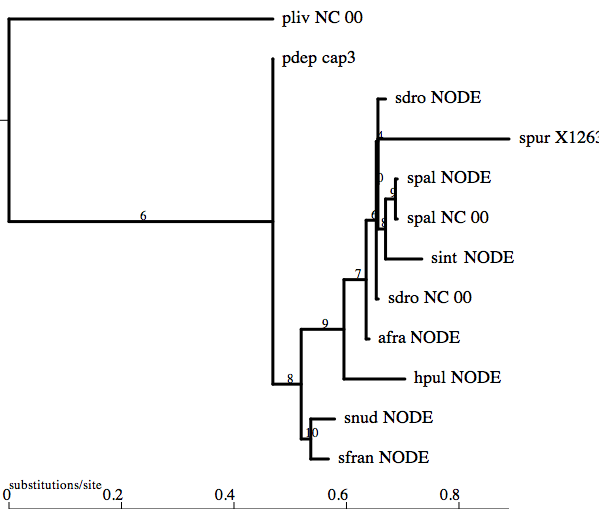

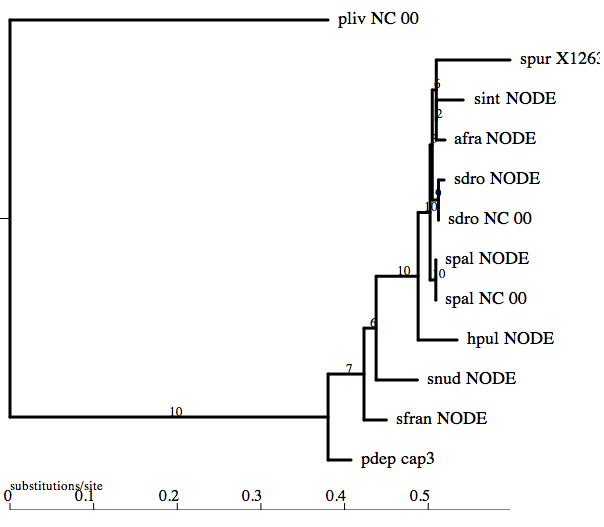

Supplement: Additional file 4: Figure S4. — Most likely ML tree for ribosomal RNA mitochondrial genes. Node support from 10 bootstrap replicates. (DOC 79 kb) [file 12862_2017_875_MOESM4_ESM.doc]
